# Functional Outcomes Across High-Risk OCT-Based Phenotypes in Intermediate Age-Related Macular Degeneration—PINNACLE Study Report 11

**DOI:** 10.1167/iovs.66.15.54

**Published:** 2025-12-17

**Authors:** Marie Louise Enzendorfer, Julia Mai, Sophie Riedl, Hrvoje Bogunović, Martin J. Menten, Daniel Rueckert, Lars G. Fritsche, A. Toby Prevost, Sobha Sivaprasad, Maximilian Pfau, Hendrik P. N. Scholl, Andrew J. Lotery, Stefan Sacu, Ursula Schmidt-Erfurth

**Affiliations:** 1 Laboratory for Ophthalmic Image Analysis, Medical University of Vienna, Vienna, Austria; 2 Department of Ophthalmology and Optometry, Medical University of Vienna, Vienna, Austria; 3 Institute of Artificial Intelligence, Centre for Medical Data Science, Medical University of Vienna, Vienna, Austria; 4 BioMedIA, Department of Computing, Imperial College London, London, United Kingdom; 5 Chair for AI in Healthcare and Medicine, Technical University of Munich, Munich, Germany; 6 Department of Biostatistics, University of Michigan, Ann Arbor, Michigan, United States; 7 Moorfields National Institute for Health and Care Biomedical Research Centre, Moorfields Eye Hospital, London, United Kingdom; 8 Institute of Ophthalmology, University College London, London, United Kingdom; 9 Institute of Molecular and Clinical Ophthalmology Basel, Basel, Basel-Stadt, Switzerland; 10 Department of Ophthalmology, University of Basel, Basel, Basel-Stadt, Switzerland; 11 Department of Clinical Pharmacology, Medical University of Vienna, Vienna, Austria; 12 Pallas Kliniken AG, Pallas Klinik Zürich, Zürich, Switzerland; 13 European Vision Institute, Basel, Basel-Stadt, Switzerland; 14 Faculty of Medicine, University of Southampton, Southampton, Hampshire, United Kingdom

**Keywords:** age-related macular degeneration, optical coherence tomography, structure–function relationships, visual function, early atrophy

## Abstract

**Purpose:**

To evaluate the association between structural optical coherence tomography (OCT) biomarkers and functional outcomes in intermediate age-related macular degeneration (iAMD) and to investigate whether stratifying eyes by OCT-based biomarkers identifies phenotypes of iAMD with impaired visual function.

**Methods:**

The baseline cohort of the PINNACLE trial underwent OCT imaging, microperimetry, best-corrected visual acuity (BCVA), and low-luminance visual acuity (LLVA) testing. OCT volumes were assessed for the presence of different morphologic features. Drusen volume and outer nuclear layer (ONL) and ellipsoid zone (EZ) thickness were quantified. Linear mixed-effect models evaluated associations between each feature and functional outcomes, including a stratification into phenotypes based on significant OCT morphology with each eye assigned to a single group.

**Results:**

This analysis included 247 eyes of 190 patients (mean age, 74.2 ± 7.4 years). The presence of subretinal drusenoid deposits (SDDs) and markers of retinal atrophy were significant contributors to lower mean retinal sensitivity (*P* < 0.05). Also, higher drusen volume and lower ONL and EZ thickness were associated with lower sensitivity. Significant changes in BCVA, LLVA, and low-luminance deficits (LLDs) were associated with increasing drusen volume and the presence of hyperreflective foci (HRF). Significant functional differences were found between individual phenotypic groups, especially highlighting functional deficit in eyes with signs of early atrophy.

**Conclusions:**

Integrating comprehensive analyses of structural OCT biomarkers with functional assessments revealed distinct phenotypic subtypes of iAMD that are associated with significant functional deficits. Particularly, early atrophy markers should be considered for patient selection and risk assessment in clinical trials and routine practice.

Age-related macular degeneration (AMD) is characterized by progressive morphological changes in the macula that exhibit substantial interindividual variability. The early and intermediate stages of AMD are primarily defined by the presence of drusen, accumulations of extracellular deposits under the retinal pigment epithelium (RPE) of the macula, and pigmentary abnormalities.^1^ Through progressive degeneration of the retinal layers and complications caused by neovascularization, patients may progress to advanced stages of AMD, posing a substantial risk of irreversible vision loss. In recent years, research efforts have been increasingly focused on developing early therapeutic interventions aimed at preventing or delaying vision loss associated with advanced AMD.^2^^–^^4^ However, to date, no specific clinical endpoints for evaluating treatment efficacy in intermediate AMD (iAMD) have been qualified by regulators.^5^

Although most commonly used AMD grading frameworks rely on structural changes observed in color fundus photography (CFP), several imaging modalities—including spectral-domain optical coherence tomography (SD-OCT)—have enabled the identification of additional pathognomonic biomarkers of iAMD or provided alternative methods for grading established abnormalities. Relevant features include drusen subtypes, subretinal drusenoid deposits (SDDs), hyperreflective foci (HRF), and early atrophic changes, which together highlight the heterogeneity of iAMD.^3^^,^^6^^,^^7^ Recently, the integration of artificial intelligence (AI)-based methods has enhanced the precision of retinal layer measurements on OCT and has shown that structural changes at the subclinical level, such as thinning of the photoreceptor layer, are valuable prognostic indicators.^8^^–^^11^ Although these structural biomarkers help assess disease severity and monitor its progression, an optimal surrogate endpoint for iAMD-related clinical trials should also reflect corresponding changes in visual function. Establishing a strong correlation between structural changes and functional outcomes is therefore essential for improving clinical management and guiding the design of future early intervention studies. Given the risk of irreversible vision loss, early detection of retinal dysfunction remains a critical priority.

Currently, best-corrected visual acuity (BCVA) is the most commonly used functional endpoint in AMD clinical trials. However, BCVA often remains relatively preserved in early and intermediate AMD because it primarily reflects foveal function and is therefore insensitive to subtle disease-related changes occurring in the para- and perifoveal regions.^12^^,^^13^ Low-luminance visual acuity (LLVA) has emerged as a more sensitive functional measure, as it has been shown to reflect non-foveal geographic atrophy more reliably than BCVA.^14^^,^^15^ Low-luminance deficits (LLD) describe the difference between visual acuity at standard luminance (BCVA) and LLVA and has been proposed as an effective functional measure in the early stages of AMD.^15^ Another promising approach is microperimetry, which measures light sensitivity across the macula while utilizing precise fundus tracking. This technique enables the detection of topographical functional changes even in early stages of AMD.^16^^,^^17^ By superimposing microperimetry test points with imaging features, derived from OCT, post hoc studies have demonstrated a reduction in retinal sensitivity overlying specific pathological features, such as higher drusen volume, SDDs, and photoreceptor degeneration.^18^^,^^19^

PINNACLE is one of the largest ongoing prospective trials investigating the progression of iAMD, offering a high-quality dataset comprised of 429 iAMD patients. A prior study by Riedl et al. (Riedl S, et al. *IOVS* 2024;65:ARVO E-Abstract 980) provided a detailed OCT-based morphologic characterization of this large baseline cohort. In this current work, we built upon these findings to comprehensively assess the impact of a broad spectrum of morphologic features on functional outcomes. The primary objective was to identify the key structural features most strongly associated with visual function deficits in iAMD. To achieve this goal, we employed a two-step analytical approach. First, we evaluated the impact of individual OCT biomarkers on retinal sensitivity, BCVA, and LLVA. Second, we developed a phenotyping framework based on the presence or absence of these features, facilitating the prediction of functional impairment and future disease progression. This phenotyping approach aimed to provide clinically relevant insights into the functional consequences of structural alterations.

## Methods

### PINNACLE Study

This study is an analysis of baseline data acquired within the scope of the prospective PINNACLE trial (NCT04269304). PINNACLE is a non-interventional, multicenter clinical study conducted at 10 collaborating sites in the United Kingdom, one in Austria, and one in Switzerland. Study protocols were approved by the East Midlands–Leicester Central Research Ethics Committee (ref. 19/EM/0163) and by the institutional ethics review boards of all participating centers. Details on trial design have previously been published by Sutton et al.^20^ Participants between the ages of 50 and 90 years with a diagnosis of iAMD, presenting with at least one druse of more than 125-µm diameter and/or pigmentary abnormalities due to AMD,^1^ were eligible for inclusion. Exclusion criteria were neovascular AMD (nAMD),^21^ as well as the presence of complete RPE and outer retinal atrophy (cRORA), defined as the presence of (1) a region of hypertransmission at least 250 µm in diameter, (2) a zone of attenuation or disruption of the RPE at least 250 µm in diameter, (3) evidence of overlying photoreceptor degeneration, and (4) absence of scrolled RPE or other signs of an RPE tear on OCT.^22^ Co-existing ocular diseases that may affect morphology or visual function were also exclusion criteria. Both eyes could be included in the study if the inclusion criteria were met. All participants provided written informed consent. The study was conducted in accordance with the tenets of the Declaration of Helsinki and adhered to the principles of Good Clinical Practice.

### OCT Analysis

SD-OCT images were acquired using SPECTRALIS HRA+OCT (Heidelberg Engineering, Heidelberg, Germany). The imaging protocol was comprised of OCT volume scans with 193 B-scans and 512 A-scans in a 20° × 20° field of view centered on the fovea. The high-speed mode with 16 frames averaged per B-scans was used.

#### Human Expert Grading

OCT volume scans were independently evaluated by two expert readers (JM, SR) for the identification of morphologic features, including SDDs, refractile drusen, hyporeflective core drusen (HCD), HRF, outer plexiform layer (OPL) subsidence, hyporeflective wedge, incomplete RPE and outer retinal atrophy (iRORA), thin and thick double layer sign (DLS), and acquired vitelliform lesions (AVLs). All images were graded by both readers, with discrepancies reviewed to achieve consensus. The morphologic requirements for the grading of feature presence are summarized in Table 1. Intergrader agreements for each structural feature, measured as percentage (%) overall agreement and Cohen's κ prior to achieving consensus, are reported in Supplementary Material S1.

**Table 1.** Grading Definitions of Structural Features. OCT Volumes Were Graded for Presence or Absence of Below Listed Features

Structural Feature	Grading Definition
Hyperreflective foci (HRF)	Well-circumscribed, hyperreflective lesions internal and detached from the RPE, with reflectivity similar to the RPE layer, thickness of at least a third of the Bruch's membrane (BM)/RPE band^6^^,^^23^
Hyporeflective core drusen (HCD)	Drusen with a content more hyporeflective than a typical druse^24^ and which do not classify as refractile druse/deposit
Hyporeflective wedge	Wedge-shaped hyporeflectivity within the area of the OPL as described within the nGA definition by Wu et al.^25^
Incomplete RPE and outer retinal atrophy (iRORA)	As defined by the CAM group: (1) a region of signal hypertransmission (HT) into the choroid; (2) a corresponding zone of attenuation or disruption of the RPE, with or without persistence of basal laminar deposits (BLamDs); and (3) evidence of overlying photoreceptor degeneration and when these criteria do not meet the definition of complete RORA (cRORA) (meaning <250 µm).^26^
Outer plexiform layer (OPL) Subsidence	Subsidence of both INL and OPL layers that exceeds the undulation to be expected with respect to underlying pathology (e.g., drusen) as described within the nGA definition by Wu et al.^25^
Refractile drusen	Pyramidal, possibly “punctate” structures at the level of the RPE; various degrees of outer retinal structure integrity and occurrence of HT and/or laminar intense hyperreflectivity at the level of the BM^23^^,^^27^
Subretinal drusenoid deposits (SDD)	Accumulations of material internal to RPE: diffuse material on the RPE extending to the EZ, mound-like on the RPE elevating the EZ, or conical projections from the RPE that may extend across the EZ^28^; a minimum of three lesions have to be present within the entire volume to be graded as SDD present
Thick and thin double layer sign (DLS)	Irregular, shallow areas of RPE elevation with clear separation of RPE and BM^29^: (a) *thick*, where the area between the RPE and BM shows multiple layers with different reflectivities, or (b) *thin*, where a single zone of low to medium reflectivity occupies the region between BM and RPE
Acquired vitelliform lesion (AVL)	Dome-shaped hyperreflective mound bounded posteriorly by the inner aspect of the RPE and anteriorly by the EZ, ELM, or outer aspect of the ONL^23^

Note that the same definitions were used by Riedl S, et al. (*IOVS* 2024;65:ARVO E-Abstract 980).

#### Automated Feature Extraction

Previously published, automated AI-based image segmentation and biomarker quantification tools were applied to OCT volumes for the quantification of retinal layer thicknesses and drusen volume.^30^^–^^35^ Ellipsoid zone (EZ) thickness was calculated based on the segmentation of the inner border of the EZ to the outer boundary of the interdigitation zone using a U-shaped convolutional neural network (CNN) architecture.^30^^,^^31^ The algorithm has previously been validated on a subset of the FILLY trial dataset of non-exudative AMD (NCT02503332).^33^ An in-house customized CNN, specialized for layer boundary regression,^36^ was used for segmentation of the outer nuclear layer (ONL). The ONL thickness was defined as the measurement from the outer border of the OPL to the external limiting membrane (ELM).^19^ Total drusen volume was quantified by segmenting the region between the outer boundary of the RPE and Bruch's membrane.^34^^,^^35^ No differentiation was made between drusen subtypes for the automatic quantification. The algorithm was previously validated on early and iAMD SPECTRALIS OCT volumes.^35^ Metrics were quantified for the full OCT volume.

### Functional Metrics

BCVA and LLVA were measured using Early Treatment of Diabetic Retinopathy Study (ETDRS) charts. Both measurements were recorded as the number of letters read. LLDs were calculated by subtracting LLVA from BCVA in the number of ETDRS letters. At the main study centers (University Hospital Southampton, Moorfields Eye Hospital, University Hospital Basel, Medical University of Vienna), patients underwent microperimetry assessments of the study eye. All study centers used the CenterVue MAIA device (iCARE, Padova, Italy) for microperimetry testing under standardized mesopic conditions. The stimulus size was set to Goldmann III (0.43° diameter). A 24-point PINNACLE standard grid (based on the central 24 points of the 10-2 pattern) was used for all baseline assessments. The grid was fovea centered and covered the central 10° (3-mm) diameter. The metric of mean retinal sensitivity (dB) was obtained by averaging the sensitivities across all test points.

### Statistical Analysis

Descriptive statistics were used to characterize the patient cohort. Associations between individual binary, as well as continuous, OCT-based structural features and functional outcomes—BCVA, LLVA, and retinal sensitivity—were assessed using univariable linear mixed-effects models (LMMs) with the functional outcomes as the dependent variable in separate LMMs. For each functional outcome, OCT features demonstrating a statistically significant effect (*P* < 0.05) were subsequently included in a multivariable LMM. This approach enabled the simultaneous evaluation of multiple contributors and their potential interactions to determine the features most strongly associated with function. To account for inter-eye correlations in bilateral patients, all statistical models included a random intercept for patient ID. Age was added to all multivariable models as a covariate. A composite binary variable was used for structural features that showed (multi-)collinearity. The model for mean retinal sensitivity was corrected for fixation stability within the central 2° range.

In a subsequent analysis, eyes were stratified into groups based on the presence of significant OCT-based features identified in the initial analysis, with each eye assigned to a single group. LMMs were employed to assess the impact of each structural phenotype on each functional outcome with post hoc testing for pairwise comparisons between the phenotypes. To account for inter-eye correlations in bilateral patients, a random intercept for patient ID was used. Age was added as a covariate. The model for mean retinal sensitivity was corrected for fixation stability within the central 2° range. Due to the exploratory nature of the analysis, no adjustment for multiple comparisons was made. Statistical analyses were conducted using SPSS Statistics 29.0.1.0 (IBM, Chicago, IL, USA). A significance level of α = 0.05 was applied throughout. All analyses are exploratory; hence, the interpretation of *P* values is descriptive.

## Results

### Cohort Characteristics

In the prospective PINNACLE trial, a total of 552 eyes of 429 patients were included at baseline. For the current analysis, only patients with complete baseline OCT data and microperimetry testing using the standardized 24-point grid were included from the respective investigating sites. Hence, the cohort for this analysis consisted of 247 eyes of 190 patients. The mean ± SD age of subjects included in this analysis was 74.2 ± 7.4 years. Of the 190 patients, 117 were female (62%). The mean retinal sensitivity of the cohort was 24.1 ± 2.4 dB. Mean BCVA and LLVA were 83.3 ± 6.4 and 67.6 ± 9.2 letters, respectively.

### Effect of OCT-Derived Structural Features on Functional Measures

Univariable and multivariable model calculations are presented in Tables 2^3^,^4^ to 5. Univariable models, correcting for inter-eye correlations, showed significant decreases in mean retinal sensitivity in the presence of HRF, SDDs, and HCD, as well as the presence of the atrophic precursors, OPL subsidence, hyporeflective wedge, and iRORA. ONL and EZ thickness demonstrated a positive correlation with mean retinal sensitivity, whereas an increased total drusen volume was associated with a decrease in mean retinal sensitivity. Feeding these variables into a multivariable LMM, SDDs and a composite binary marker consisting of atrophic precursors (OPL subsidence/wedge/iRORA) remained associated with significantly lower mean retinal sensitivity (−0.8 dB, *P* = 0.006; −1.0 dB, *P* = 0.002, respectively). Also, higher drusen volume and lower ONL and EZ thickness were associated with decreases in sensitivity (−3.2 dB/mm^3^, *P* < 0.001; −0.1 dB/µm, *P* = 0.031; −0.1 dB/µm, *P* = 0.043, respectively). BCVA showed significant changes in the presence of HRF and with respect to total drusen volume. However, in the multivariable LMM, only total drusen volume was associated with lower BCVA (−6.0 letters/mm^3^, *P* = 0.017). LLVA showed more significant associations with individual features including HRF, iRORA, HCD, ONL thickness, EZ thickness, and total drusen volume. However, the multivariable LMM indicated that HRF presence (−3.7 letters, *P* = 0.003) and total drusen volume (−14.2 letters/mm^3^, *P* < 0.001) were the most significant contributors to LLVA decrease. LLDs were shown to significantly increase with the presence of HRF and higher total drusen volume (2.8 letters, *P* = 0.004; 7.8 letters/mm^3^, *P* = 0.010, respectively). The univariable and multivariable models showed that thin and thick DLS, refractile drusen, and AVLs were not associated with visual function deficits. HCD were associated with lower mean retinal sensitivity and LLVA in the univariable analysis, but were no longer associated when correcting for co-existing features. The calculated models also indicated the significant impact of age on functional outcomes.

**Table 2.** Univariable and Multivariable Mixed-Effect Models Showing the Effect of Different Morphological Variables on Mean Retinal Sensitivity (dB)

		Univariable		Multivariable	
Feature	*N*	Estimate (95% CI)	*P*	Estimate (95% CI)	*P*
HRF	164	−1.3 (−1.8 to −0.7)	**<0.001**	−0.3 (−0.8 to 0.3)	0.332
SDDs	101	−1.0 (−1.7 to −0.4)	**0.002**	−0.8 (−1.3 to −0.2)	**0.006**
Thick DLS	7	−0.3 (−2.0 to 1.3)	0.703	—	—
Thin DLS	19	−0.7 (−1.8 to 0.4)	0.238	—	—
OPL subsidence	41	−1.5 (−2.4 to −0.7)	**<0.001**	—	—
Wedge	14	−2.6 (−4.0 to −1.3)	**<0.001**	—	—
iRORA	45	−1.5 (−2.2 to −0.8)	**<0.001**	—	—
OPL subsidence/wedge/iRORA^*^	60	−1.6 (−2.2 to −0.9)	**<0.001**	−1.0 (−1.6 to −0.4)	**0.002**
Refractile drusen	19	−1.0 (−2.1 to 0.2)	0.096	—	—
HCD	42	−1.0 (−1.8 to −0.2)	**0.011**	−0.3 (−1.0 to 0.3)	0.325
AVLs	13	−0.1 (−1.3 to 1.1)	0.882	—	—
ONL thickness (µm)	247	0.1 (0.1 to 0.2)	**<0.001**	0.1 (0.0 to 0.1)	**0.031**
EZ thickness (µm)	247	0.4 (0.2 to 0.5)	**<0.001**	0.1 (0.0 to 0.3)	**0.043**
Drusen volume (mm^3^)	247	−3.8 (−5.7 to −2.0)	**<0.001**	−3.2 (−5.0 to −1.5)	**<0.001**
Age (y)	247	−0.1 (−0.2 to −0.1)	**<0.001**	−0.1 (−0.1 to −0.0)	**<0.001**
Fixation stability 2° (%)	247	0.0 (0.0 to 0.0)	**0.003**	0.0 (0.0 to 0.0)	**0.004**

AVL, acquired vitelliform; CI, confidence interval; DLS, double-layer sign; EZ, ellipsoid zone; HCD, hyporeflective core drusen; HRF, hyperreflective foci; iRORA, incomplete RPE and outer retinal atrophy; N, number of eyes with variable present; ONL, outer nuclear layer; OPL, outer plexiform layer; SDD, subretinal drusenoid deposits.

Estimates for binary variables indicate the predicted mean retinal sensitivity change in the presence of the variable. *N* represents the number of eyes with that variable present. Bold *P*-values indicate statistical significance.

* Composite variable of atrophy precursors created due to multicollinearity in the multivariable model.

**Table 3.** Univariable and Multivariable Mixed-Effect Models Showing the Effect of Different Morphological Variables on BCVA (Letters)

		Univariable		Multivariable	
Feature	*N*	Estimate (95% CI)	*P*	Estimate (95% CI)	*P*
HRF	164	−2.4 (−4.0 to −0.7)	**0.005**	−1.5 (−3.1 to 0.2)	0.089
SDDs	101	−0.7 (−2.4 to 0.1)	0.410	—	—
Thick DLS	7	0.1 (−3.8 to 5.7)	0.680	—	—
Thin DLS	19	1.0 (−2.0 to 4.0)	0.518	—	—
OPL subsidence	41	0.2 (−1.9 to 2.4)	0.823	—	—
Wedge	14	0.6 (−2.9 to 4.1)	0.738	—	—
iRORA	45	−2.0 (−4.0 to 0.1)	0.059	—	—
Refractile drusen	19	−0.7 (−3.8 to 2.3)	0.636	—	—
HCD	42	−1.1 (−3.2 to 1.1)	0.332	—	—
AVLs	13	−2.9 (−6.4 to 0.1)	0.104	—	—
ONL thickness (µm)	247	0.1 (−0.1 to 0.2)	0.338	—	—
EZ thickness (µm)	247	0.3 (−0.1 to 0.7)	0.118	—	—
Drusen volume (mm^3^)	247	−5.2 (−10.3 to −0.22)	**0.041**	−6.0 (−11.0 to −1.1)	**0.017**
Age (y)	247	−0.3 (−0.4 to −0.2)	**<0.001**	−0.3 (−0.4 to −0.2)	**<0.001**

Estimates for binary variables indicate the predicted BCVA change in the presence of the variable. The composite variable OPL subsidence/wedge/iRORA is not included in this multivariable model because no atrophic precursors showed significance in the univariable models. *N* represents the number of eyes with that variable present. Bold *P*-values indicate statistical significance.

**Table 4.** Univariable and Multivariable Mixed-Effect Models Showing the Effect of Different Morphological Variables on LLVA (Letters)

		Univariable		Multivariable	
Feature	*N*	Estimate (95% CI)	*P*	Estimate (95% CI)	*P*
HRF	164	−6.0 (−8.3 to −3.7)	**<0.001**	−3.7 (−6.2 to −1.3)	**0.003**
SDDs	101	−1.2 (−3.7 to 1.3)	0.335	—	—
Thick DLS	7	2.5 (−4.3 to 9.3)	0.466	—	—
Thin DLS	19	1.2 (−3.2 to 5.6)	0.600	—	—
OPL subsidence	41	−0.1 (−3.3 to 3.0)	0.929	—	—
Wedge	14	2.3 (−2.7 to 7.2)	0.374	—	—
iRORA	45	−3.7 (−6.7 to −0.8)	**0.012**	−0.8 (−3.7 to 2.1)	0.580
Refractile drusen	19	−3.1 (−7.5 to 1.2)	0.159	—	—
HCD	42	−3.1 (−6.2 to 0.0)	**0.047**	−1.0 (−3.8 to 1.9)	0.497
AVLs	13	−4.7 (−9.7 to 0.3)	0.066	—	—
ONL thickness (µm)	247	0.2 (0.0 to 0.4)	**0.019**	0.1 (−0.1 to 0.2)	0.565
EZ thickness (µm)	247	0.7 (0.2 to 1.2)	**0.007**	0.1 (−0.4 to 0.6)	0.691
Drusen volume (mm^3^)	247	−16.0 (−22.9 to −9.0)	**<0.001**	−14.2 (−21.2 to −7.2)	**<0.001**
Age (y)	247	−0.4 (−0.5 to −0.2)	**<0.001**	−0.4 (−0.5 to −0.2)	**<0.001**

Estimates for binary variables indicate the predicted LLVA change in the presence of the variable. The composite variable OPL subsidence/wedge/iRORA is not included in this multivariable model because only iRORA was significant. *N* represents the number of eyes with that variable present. Bold *P*-values indicate statistical significance.

**Table 5.** Univariable and Multivariable Mixed-Effect Models Showing the Effect of Different Morphological Variables on LLD (Letters)

		Univariable		Multivariable	
Feature	*N*	Estimate (95% CI)	*P*	Estimate (95% CI)	*P*
HRF	164	3.6 (1.7 to 5.4)	**<0.001**	2.8 (0.9 to 4.7)	**0.004**
SDDs	101	0.0 (−1.9 to 2.0)	0.971	—	—
Thick DLS	7	−1.8 (−7.1 to 3.4)	0.489	—	—
Thin DLS	19	3.3 (−0.0 to 6.6)	0.053	—	—
OPL subsidence	41	0.0 (−2.5 to 2.5)	0.979	—	—
Wedge	14	−1.9 (−5.8 to 2.1)	0.351	—	—
iRORA	45	1.1 (−1.2 to 3.4)	0.347	—	—
Refractile drusen	19	1.2 (−1.6 to 5.4)	0.283	—	—
HCD	42	1.5 (−1.0 to 3.9)	0.238	—	—
AVLs	13	1.4 (−2.5 to 5.3)	0.572	—	—
ONL thickness (µm)	247	0.1 (0.0 to 0.3)	0.057	—	—
EZ thickness (µm)	247	0.2 (−0.2 to 0.7)	0.259	—	—
Drusen volume (mm^3^)	247	9.4 (−15.1 to −3.7)	**0.001**	7.8 (1.9 to 13.6)	**0.010**
Age (y)	247	−0.1 (−0.2 to 0.0)	0.140	0.1 (0.0 to 0.3)	0.112

Estimates for binary variables indicate the predicted LLD change in the presence of the variable. *N* represents the number of eyes with that variable present. Bold *P*-values indicate statistical significance.

### Functional Outcomes for Progressive Severity Levels in iAMD Phenotypes

**Figure 1.**
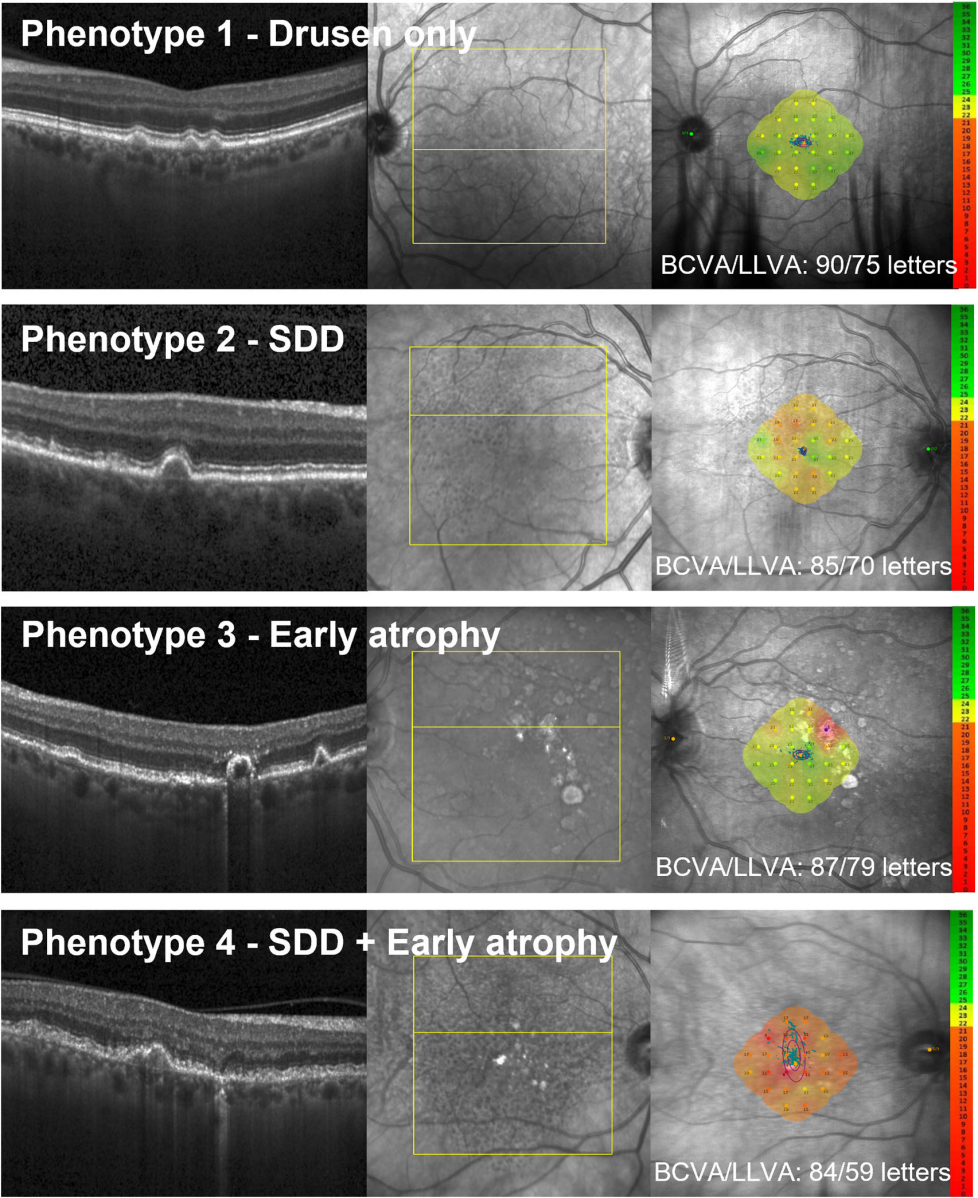
Example eyes for the structurally defined phenotypic groups with their corresponding 24-point microperimetry grid. Each eye was assigned to a single group. The phenotypes were defined as phenotype 1, drusen only; phenotype 2, SDDs; phenotype 3, early atrophy; and phenotype 4, SDDs+early atrophy. The *far right* shows the en face image with the 24-point microperimetry grid placed centrally on the fovea. BCVA and LLVA for the eye are given in letters.

**Figure 2.**
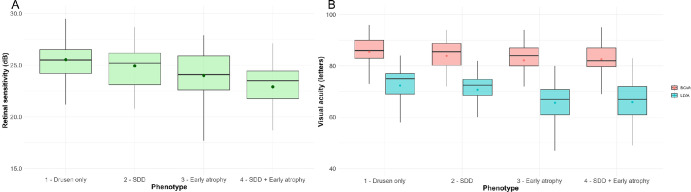
Retinal sensitivity (**A**) and BCVA and LLVA (**B**) for the four phenotypic groups. Box plots summarize the distribution of outcome variables across the four phenotypic groups. The mean value is indicated by the point.

Based on the preceding analysis of how structural features influence functional outcomes, eyes were stratified into one of four phenotypic groups, incorporating structural features that were significant in the multivariable LMMs (*P* < 0.05). Each eye was assigned to a single group based on its most advanced morphologic grading: (1) eyes with drusen only (phenotype 1, drusen only); (2) eyes with SDDs but no signs of early atrophy (phenotype 2, SDDs); (3) eyes with signs of early atrophy defined by presence of HRF and/or iRORA and/or OPL subsidence and/or hyporeflective wedge but no SDDs (phenotype 3, early atrophy); and (4) eyes with both SDDs and signs of early atrophy (phenotype 4, SDDs+early atrophy). Examples of stratified eyes are presented in Figure 1. A descriptive analysis of the defined groups is given in Table 6 and Figure 2.

**Table 6.** Descriptive Statistics of the Phenotypic Groups

Variable	Phenotype 1, Drusen Only	Phenotype 2, SDDs	Phenotype 3, Early Atrophy	Phenotype 4, SDDs+Early Atrophy
Eyes, *n* (%)	60 (24)	22 (9)	85 (35)	80 (32)
Sensitivity (dB), mean ± SD	25.5 ± 1.8	24.9 ± 2.2	24.0± 2.8	22.9± 2.4
BCVA (letters), mean ± SD	85.5 ± 6.2	84.0 ± 6.8	82.1 ± 6.9	82.6 ± 5.7
LLVA (letters), mean ± SD	72.4 ± 7.5	70.7 ± 7.7	64.9 ± 10.2	65.9 ± 8.3
LLD (letters), mean ± SD	13.1 ± 4.1	13.2 ± 3.3	17.2 ± 6.8	16.7 ± 5.8
Age (y), mean ± SD	72.4 ± 6.9	74.9 ± 7.2	73.5 ± 7.8	76.0 ± 7.3

Pairwise comparisons using LMMs correcting for inter-eye correlations and age were carried out (Table 7). Significant differences in mean retinal sensitivity were found between the following structurally defined groups: Drusen only:Early atrophy [1.0 dB, *P* = 0.003], Drusen only:SDD + Early atrophy [1.9 dB, *P* < 0.001], SDD:SDD + Early atrophy [1.1 dB, *P* = 0.015], Early atrophy:SDD + Early atrophy [0.8 dB, *P* = 0.019]. BCVA showed significant differences between the phenotypic group with only drusen and the group with signs of early atrophy (2.6 letters, *P* = 0.012).

**Table 7.** Pairwise Comparison of Differences in Functional Outcomes (Mean Retinal Sensitivity, BCVA, LLVA, and LLD) Among the Four Phenotypic Groups Using Linear-Mixed Effect Models

	Mean Retinal Sensitivity (dB)		BCVA (Letters)		LLVA (Letters)		LLD (Letters)	
Phenotype	Mean Difference (95% CI)	*P*	Mean Difference (95% CI)	*P*	Mean Difference (95% CI)	*P*	Mean Difference (95% CI)	*P*
1								
2	0.7 (−0.2 to 1.7)	0.136	0.8 (−2.2 to 3.8)	0.604	0.7 (−3.5 to 4.9)	0.750	0.3 (−3.0 to 3.7)	0.842
3	1.0 (0.4 to 1.7)	**0.003**	2.6 (0.6 to 4.7)	**0.012**	6.6 (3.7 to 9.4)	**<0.001**	−4.1 (−6.4 to −1.7)	**<0.001**
4	1.9 (1.1 to 2.6)	**<0.001**	1.9 (−0.2 to 4.0)	0.082	5.1 (2.2 to 8.0)	**<0.001**	−2.8 (−5.3 to −0.4)	**0.024**
2								
1	−0.7 (−1.7 to 0.2)	0.136	−0.8 (−3.8 to 2.2)	0.604	−0.7 (−4.9 to 3.5)	0.750	−0.3 (−3.7 to 3.1)	0.842
3	0.3 (−0.7 to 1.2)	0.543	1.8 (−1.0 to 4.7)	0.211	5.9 (1.9 to 9.9)	**0.004**	−4.4 (−7.7 to −1.1)	**0.009**
4	1.1 (0.2 to 2.0)	**0.015**	1.1 (−1.8 to 3.9)	0.457	4.4 (0.4 to 8.4)	**0.030**	−3.2 (−6.4 to 0.0)	0.053
3								
1	−1.0 (−1.7 to −0.4)	**0.003**	−2.6 (−4.7 to −0.6)	**0.012**	−6.6 (−9.4 to −3.7)	**<0.001**	4.1 (1.7 to 6.4)	**<0.001**
2	−0.3 (−1.2 to 0.7)	0.543	−1.8 (−4.7 to 1.0)	0.211	−5.9 (−9.9 to −1.9)	**0.004**	4.4 (1.1 to 7.7)	**0.009**
4	0.8 (0.1 to 1.5)	**0.019**	−0.8 (−2.7 to 1.2)	0.440	−1.5 (−4.2 to 1.2)	0.272	1.3 (−1.0 to 3.5)	0.278
4								
1	−1.9 (−2.6 to −1.1)	**<0.001**	−1.9 (−4.0 to 0.2)	0.082	−5.1 (−8.0 to −2.2)	**<0.001**	2.8 (0.4 to 5.3)	**0.024**
2	−1.1 (−2.0 to −0.2)	**0.015**	−1.1 (−3.9 to 1.8)	0.380	−4.4 (−8.4 to −0.4)	**0.030**	3.2 (0.0 to 6.4)	**0.050**
3	−0.8 (−1.5 to −0.1)	**0.019**	0.8 (−1.2 to 2.7)	0.440	1.5 (−1.2 to 4.2)	0.272	−1.3 (−3.5 to 1.0)	0.278

In terms of LLVA outcomes, phenotypes 1 and 2 had significantly higher LLVA values than phenotypes with signs of early atrophy: Drusen only:Early atrophy [6.6 letters, *P* < 0.001], Drusen only:SDD + Early atrophy [5.1 letters, *P* < 0.001], SDD:Early atrophy [5.9 letters, *P* = 0.004], SDD:SDD + Early atrophy [4.4 letters, *P* = 0.030]. These differences were also reflected in LLDs. No statistically significant differences were found between the group with only drusen (phenotype 1) and the SDD group (phenotype 2).

## Discussion

In recent years, clinical trials have increasingly highlighted the importance of structure–function correlations in macular diseases.^37^ This study provides a comprehensive state-of-the-art analysis incorporating a broad range of OCT-based structural features to identify the most influential contributors to functional decline in iAMD during the earliest pathophysiological processes. The large sample size and standardized high-quality imaging data of a prospective trial further strengthen the reliability of our findings. Additionally, our strategic phenotyping framework underscores distinct severity variations within iAMD, revealing significant functional loss even at this intermediate stage.

Consistent with previous studies, our analysis identified microperimetry-derived visual function outcomes as the most closely linked to structural morphology. As anticipated, BCVA demonstrated the weakest association with types and levels of structural changes in iAMD. In our multivariate model, significant loss in BCVA was observed only in association with increased drusen volume and failed to discriminate functional differences among phenotypic subgroups that were detected by microperimetry and LLVA. Phenotypes exhibiting reduced LLVA also demonstrated greater LLDs, underscoring pathophysiological changes that remain undetected by standard high-contrast visual acuity testing.

Total drusen volume was shown to be a significant contributor to all measures of visual function. This is in line with previous analyses by different groups.^38^^–^^43^ As a hallmark feature of AMD, drusen volume is known to be highly prognostic of progression and conversion to late-stage AMD.^44^^–^^46^ The major cause for this immediate impact on central visual function is the central location of drusen deposition preferentially in the foveal region.^32^ Drusen volume, however, can fluctuate over time, and studies have shown that drusen regression does not always lead to atrophy.^47^ Considering this, drusen volume by itself may not be an ideal surrogate marker for use in clinical trials.

Multiple studies have identified SDDs as a major risk factor for AMD progression and a potentially critical phenotype in AMD^48^^,^^49^; however, their impact on function shows inconsistent findings.^50^^–^^53^ Histological analyses have linked this structural feature to greater dysfunction of the RPE,^54^ and multimodal imaging studies have indicated its association with photoreceptor degeneration.^49^^,^^55^^,^^56^ Importantly, SDDs have been shown to modify treatment effects in iAMD.^57^^,^^58^ Our analysis confirmed a significant association between SDD presence and reduced mean retinal sensitivity,^59^ likely attributable to photoreceptor and especially rod dysfunction across the macula in eyes presenting with SDDs.^19^^,^^51^^,^^60^^–^^62^ However, our findings did not indicate significant BCVA or LLVA loss in their presence, suggesting that these measures are less sensitive to this structural feature. This may be explained by the predominantly parafoveal and perifoveal distribution of SDDs, where functional impairment is less likely to be captured by standard central visual acuity assessments. The foveal sparing effect of SDDs has been shown by a range of different imaging modalities,^6^^,^^63^^,^^64^ as well as histological studies.^54^

Atrophic precursors examined in our study included iRORA, as well as features of nascent geographic atrophy (nGA), OPL subsidence, and hyporeflective wedge. These OCT markers are highly predictive of progression to GA, underscoring their relevance in risk stratification at the intermediate stage of AMD.^65^^,^^66^ In our analysis, early atrophic markers emerged as the strongest contributors to mean retinal sensitivity deficits, highlighting their association with overall photoreceptor health beyond a location-specific effect. LLVA and BCVA did not reflect functional changes associated with these atrophic precursors, likely due to their predominantly parafoveal location as demonstrated by Saßmannshausen et al. and by Wu et al.^6^^,^^27^ Interestingly, the presence of HRF, representing migrating RPE cells,^67^^,^^68^ was not a main contributor to mean retinal sensitivity but impacted LLVA significantly. This may be attributed to the small size of HRF, limiting their detection within microperimetry testing. Consistently, pixelwise co-registration analyses have demonstrated a significant association between HRF volume and retinal sensitivity.^19^^,^^42^ Previous studies showed HRF to be predominantly located within the center of the macula, explaining their impact on LLVA outcomes.^6^ Their impact on LLVA is in concordance with a recent study by Liu et al.^38^

Our study incorporated AI-based measures of subclinical changes, further elucidating the role of photoreceptor layer alterations in functional decline. Histological studies have demonstrated progressive ONL and EZ thinning with disease progression toward GA.^69^ Furthermore, AI-driven analyses have suggested that EZ attenuation precedes RPE loss and represents one of the earliest anatomical changes preceding macular atrophy.^66^^,^^70^^,^^71^ In our analysis, both EZ thickness and ONL thickness were inversely associated with reduced mean retinal sensitivity. This is in agreement with current publications that have reported a point-wise association between EZ thickness and ONL thickness with microperimetry-measured sensitivity.^19^^,^^72^ It is also in line with work from Roh et al.,^73^ who previously demonstrated the strong association with mean retinal sensitivity in the presence of other confounding factors and coexisting OCT features. In our univariable analysis, EZ and ONL thickness also proved to be associated with LLVA, but this effect was no longer present when accounting for other structural markers in the multivariable model. Previous histological studies have demonstrated the increased vulnerability of rods over cones,^74^ providing a possible explanation for why functional assessments centered on the cone-dominated fovea often remain unaffected until later disease stages. In contrast, mesopic testing conditions in microperimetry capture rod-mediated signals and can reveal subtle sensitivity losses at an earlier stage.^19^ Structural alterations such as photoreceptor inner and outer segment thinning^72^^,^^75^^,^^76^ or reduced photopigment regeneration^77^ may also reduce retinal sensitivity well before high-contrast, cone-mediated visual acuity is affected.

Our phenotypic stratification based on the presence of key structural OCT markers supports the hypothesis that iAMD encompasses distinct subgroups with differing functional impairments, likely reflective of the temporal disease sequence. Especially the presence of early atrophic markers (HRF, iRORA, hyporeflective wedge, and OPL subsidence) revealed a significant impact on all analyzed visual outcomes. Only microperimetry revealed that eyes exhibiting both SDDs and early atrophic markers (phenotype 4, SDDs+early atrophy) showed the greatest overall loss in retinal sensitivity, consistent with a more severe, globally impaired disease state. In contrast, the presence of SDDs alone did not distinguish patients functionally from those with drusen only, suggesting that, although SDDs contribute to decreased retinal sensitivity, their presence alone does not define a patient group with lower function. Nevertheless, the frequent co-occurrence of SDDs with early atrophic features in our cohort supports the view that SDDs may serve as an indicator of an ongoing, slowly progressing atrophic process. This observation aligns with prior work by Kumar et al.,^51^ proposing that the overall extent of SDDs within an eye may be an indicator of broader pathogenic changes contributing to visual deficits.

Various studies have demonstrated the ability of LLVA and microperimetry to differentiate functional impairment across different AMD stages compared to healthy controls.^78^^–^^81^ However, these studies have also shown that functional variation within stages suggests that retinal function is not convincingly dependent on the commonly used classification based on conventional CFP gradings.^78^^,^^82^ The associations seen between our defined OCT-based phenotypic groups of iAMD and function, especially retinal sensitivity, underscore the benefit of incorporating morphologies seen on OCT into stratification approaches. Given that current interventional trials for iAMD^2^^,^^83^ largely rely on drusen-based anatomical endpoints, our findings strongly suggest that drusen alone may be insufficient for patient stratification. Early markers of atrophy play an important role in progressive retinal dysfunction. Future clinical trials should benefit from incorporating structural features beyond drusen to refine eligibility criteria and outcome measures to reflect functional differences better. Additionally, our study supports the inclusion of mean retinal sensitivity as a functional endpoint in iAMD research.

Several limitations must be acknowledged. The cross-sectional nature of this analysis precludes definitive conclusions on the predictive value of the identified biomarkers or phenotypic stratification for functional changes and late-stage AMD development. Longitudinal studies are necessary to assess their long-term impact on functional decline. Furthermore, it is important to note that functional testing remains inherently subjective, with potential variability in a multicenter setting. Moreover, although our study employed detailed retinal grading by expert graders, the binary classification of structural markers may overlook severity and spatial extent. Future research should explore quantitative approaches for enhanced precision in assessing structure–function relationships in iAMD. Additionally, our study employed a two-step statistical approach, where phenotypic groups were stratified based on statistical analysis of the same dataset. To strengthen the validity of our findings, future studies will replicate these group comparisons using an independent iAMD dataset. Furthermore, as multimodal imaging offers additional insights into disease severity beyond the scope of the current analysis, future studies should integrate modalities such as CFP to enable a more comprehensive, multimodal characterization of iAMD phenotypes.

## Supplementary Material

Supplement 1
