# Supplementary material for: Functional Outcomes Across High-Risk OCT-Based Phenotypes in Intermediate Age-Related Macular Degeneration—PINNACLE Study Report 11
Source: Invest Ophthalmol Vis Sci. 2025 Dec 17;66(15):54. doi: 10.1167/iovs.66.15.54 (PMC12716453; doi:10.1167/iovs.66.15.54)
Supplement: Supplement 1 [file iovs-66-15-54_s001.pdf]

Supplement 1

Table S1. Inter-grader agreement for the presence of structural features on optical coherence tomography (OCT) prior to reaching consensus. *Interpretation of Cohen's Kappa coefficient is based on the definitions by Landis JR. & Koch GG., 1977.*[1]

Feature presence	n	% Overall agreement	Kappa (95% CI)	Agreement
Hyperreflective Foci (HRF)	247	87%	0.78 (0.65-0.90)	Substantial
Hyporeflective Core Drusen (HCD)	247	86%	0.49 (0.38-0.60)	Moderate
Hyporeflective Wedge	247	96%	0.59 (0.46-0.71)	Moderate
Incomplete retinal pigment epithelium (RPE) and outer retinal atrophy (iRORA)	247	79%	0.48 (0.37-0.59)	Moderate
Outer Plexiform Layer (OPL) Subsidence	247	84%	0.48 (0.38-0.59)	Moderate
Refractile drusen	247	92%	0.43 (0.31-0.55)	Moderate
Subretinal drusenoid deposits (SDD)	247	90%	0.81 (0.68-0.93)	Almost perfect
Thick Double Layer Sign (thickDLS)	247	97%	0.45 (0.32-0.57)	Moderate
Thin Double Layer Sign (thinDLS)	247	91%	0.39 (0.26-0.51)	Fair
Acquired Vitelliform Lesion (AVL)	247	89%	0.35 (0.25-0.46)	Fair

Reference

1. Landis JR, Koch GG. The Measurement of Observer Agreement for Categorical Data. *Biometrics*. 1977;33(1):159. doi:10.2307/2529310
